# Anti‐TNFα induced lupus due to infliximab therapy in a patient with concurrent Crohn's disease

**DOI:** 10.1002/ccr3.7673

**Published:** 2023-07-18

**Authors:** Akanksha Sharma, Taha Ahmed, Aashna Mehta, Julius Birnbaum, Abhigan Babu Shrestha

**Affiliations:** ^1^ Department of Internal Medicine UPMC Mercy Pittsburg Pennsylvania USA; ^2^ Faculty of Medicine University of Debrecen Debrecen Hungary; ^3^ Department of Internal Medicine M Abdur Rahim Medical College Dinajpur Bangladesh

**Keywords:** antinuclear antibodies, anti‐TNFa inhibitor‐induced lupus, Crohn's disease

## Abstract

Anti‐TNFα inhibitor‐induced Lupus (ATIL) is a rare syndrome characterized by a wide array of symptoms ranging from skin manifestations to organ‐specific symptoms such as pleural effusions, pericardial effusions, hepatotoxicity, etc. Infliximab is implicated in most cases, and ATIL usually develops between the first month and 4 years of infliximab application. In this report, we present an interesting case of ATIL that developed rather gradually upon anti‐TNFa used to treat Crohn's disease. The patient presented with oral ulcers, photosensitive rash, diffuse alopecia, inflammation of the hands, and pleuritic chest pain. Comprehensive serological testing revealed the presence of antinuclear antibodies and anti‐DNA antibodies. During the evaluation, the patient developed headaches, followed by a brain MRI that suggested nonspecific white matter lesions. Given the chronic development of symptoms, invasive examinations such as an arteriogram were performed to exclude CNS vasculitis, which showed no evidence of the vasculitis. Therefore, in the absence of any clear radiographical signs of vasculitis, patients should not undergo invasive studies, including an angiogram. The CNS angiogram can be associated with several side effects, including damage to the blood vessel, bruising or bleeding at the puncture site, and infection, which can further aggravate ATIL. Although rare, ATIL should always be considered while evaluating a patient with suggestive symptoms on infliximab therapy. Further research on identifying the variety of clinical presentations of ATIL and the underlying pathophysiology can help improve health policy and clinical practice by reducing unnecessary examination and allowing early management and better‐quality care.

## 1 INTRODUCTION

Infliximab causes most antitumor necrosis factor‐α‐induced lupus cases (ATIL), followed by etanercept and adalimumab. ATIL is characterized by a wide array of symptoms ranging from common, minor cutaneous lesions to uncommon, dangerous pleural or pericardial effusions, demyelination, hepatotoxicity, and malignancy are just a few symptoms. Constitutional symptoms frequently accompany a positive autoantibody serology. If symptoms and antitumor necrosis factor (TNF) therapy occur concurrently and at least one serologic and one non‐serologic American College of Rheumatology criteria are met, a confirmative diagnosis can be made. Before starting anti‐TNF medication, baseline immunological tests (including those for antinuclear antibodies [ANA]) must be performed, and patients should be closely monitored for the emergence of lupus symptoms. If symptoms develop, the implicated drug should be discontinued, followed by resolution of symptoms generally between 3 weeks and 6 months.^1^, ^2^ Many individuals might still require standard therapy for idiopathic SLE in addition to stopping their anti‐TNF medication from relieving the symptoms and ensuring patient well‐being fully. This case report was drafted in line with the CARE guideline.^3^

## 2 CASE PRESENTATION

A 56‐year‐old gentleman presented for evaluation of white‐matter lesions in the context of TNF‐induced systemic lupus erythematosus (SLE). Medical history includes Crohn's disease, diagnosed 22 years prior to evaluation (PTE) at the same medical facility. His Crohn's was refractory to non‐biological disease‐modifying anti‐rheumatic disease drugs (DMARDS). Subsequently, he was treated with infliximab (5 mg/kg), which proved effective and resulted in disease remission. Subsequently, on a 1‐year follow‐up, he developed oral ulcers, photosensitive rash, diffuse alopecia, inflammation of the hands, and pleuritic chest pain.

Further serological testing revealed the presence of ANA at a titer of 1:320 in a homogeneous pattern; anti‐double strand DNA (419 Units). He did not have evidence for antinuclear extractable antigen (anti‐ENA) and complement proteins C3 and C4 were normal. A brain MRI was prompted by mild headaches, which revealed nonspecific white‐matter lesions. Further serological workup demonstrated no evidence for antiphospholipid antibodies: with no evidence for anti‐cardiolipin antibodies, anti‐beta‐2 glycoprotein antibodies, or lupus anticoagulant. Central nervous (CNS) vasculitis in the context of tumor necrosis factor inhibitors was considered as a differential. However, lumbar puncture studies were normal (zero white blood cells, no oligoclonal bands), and a formal four‐vessel angiogram did not show evidence of CNS vasculitis, thus, effectively ruling it out.

Further management included discontinuation of infliximab, and the patient was then referred to our clinic. He was started on hydroxychloroquine 400 mg which led to complete remission of lupus symptoms, and further testing showed that he was now seronegative with regard to ANA and anti‐double strand DNA antibodies. During this time, the patient had a flare of Crohn's disease and was started on 45 mg of ustekinumab, which led to the resolution of his Crohn's disease.

## 3 DISCUSSION

We present a case of a TNF‐inhibitor‐induced lupus syndrome, which was interesting in many regards. One aspect was the chronic onset, which is quite unusual for a TNF‐inhibitor‐induced lupus syndrome to present after being on a stable dose of a TNF‐inhibitor agent for 16 years to maintain remission. Typically, patients who develop ATIL develop symptoms anywhere from over a month to 4 years on anti‐TNF therapy.^4^ Moreover, the pathophysiology of anti‐TNF in the emergence of SLE remains unclear, and several theories have attempted to explain the prevalence of lupus or lupus‐like symptoms in patients receiving anti‐TNF medication. For instance, anti‐TNFinhibits the production of Th1 cytokines, shifting the immune response toward the production of Th2 cytokines, IL‐10, and IFN‐, as per the “cytokine shift” theory.^5^, ^6^ Another theory is based on the idea that systemic TNF suppression could prevent apoptosis by reducing CD44 expression. This can influence phagocytosis and the removal of nuclear debris and apoptotic neutrophils, encouraging the formation of autoantibodies against DNA and other nuclear antigens.^5^ In the context of the varied theories, the exact pathophysiology remains debatable, which, if further explored, can possibly provide insight into the difference in onset observed in a complex case such as this patient. The lack of precise clinical criteria for the diagnosis of ATIL makes the diagnosis more challenging.^5^

Clinical features vary from usual SLE like symptoms to very unusual clinical features. Almoallim et al. analyzed 33 case reports of such disease which showed a wide spectrum of features like rash, polysynovitis, fever, myalgias, pleural effusion, pericardial effusion, nephritis, valvulitis, pneumonitis, and deep vein thrombosis in decreasing manner of prevalence.^4^

In addition, this patient's MRI lesions were interpreted to reflect CNS vasculitis, and he subsequently underwent invasive studies, including lumbar puncture studies and a central nervous system angiogram. However, such an invasive study was unwarranted for several reasons. First, although TNF‐induced lupus primarily causes musculoskeletal and cutaneous manifestations (as seen in our patient), neurological lesions in TNF‐induced lupus are exceedingly unusual and nonspecific. Studies have shown that the prevalence of CNS vasculitis in lupus is extremely rare (<1%). Furthermore, the most common presentations of CNS vasculitis are encephalopathy, severe headaches, and stroke‐like symptoms. For example, the most important brain findings in CNS vasculitis on MRI vary from small ischemic changes to frank infarction, hemorrhage, and white matter edema and may show contrast material enhancement. The cerebral arteries may demonstrate a beaded appearance with variable degrees of stenosis, occlusion, and contrast enhancement of the vessel wall. Therefore, in the absence of any clear radiographical signs of vasculitis, patients should not undergo invasive studies, including an angiogram. The CNS angiogram can be associated with several side effects, including damage to the blood vessel, bruising or bleeding at the puncture site, and infection, which can further aggravate ATIL. Although rare, ATIL should always be considered and evaluated utilizing serology and clinical presentation while managing a patient with suggestive symptoms on infliximab therapy.

## 4 CONCLUSION

In summary, we report on a notable case of 16 years of TNF‐inhibitor before lupus‐like syndrome. An ANA and anti‐DNA reference assay should be performed before starting anti‐TNF alpha. Lupus syndromes and serologies respond to discontinuation of offending TNF alpha inhibitors, and Plaquenil can lead to a resolution of symptoms, thereby reducing unnecessary and time‐consuming invasive interventions such as angiograms.

## AUTHOR CONTRIBUTIONS

**Akanksha sharma:** Supervision; writing – review and editing. **taha ahmed:** Writing – original draft. **Aashna Mehta:** Writing – original draft. **julius Birnbaum:** Writing – original draft; writing – review and editing. **Abhigan Babu Shrestha:** Writing – review and editing.

## FUNDING INFORMATION

Authors have no funding to declare.

## CONFLICT OF INTEREST STATEMENT

Authors have no conflicts of interest to declare.

## ETHICAL APPROVAL AND CONSENT TO PARTICIPATE

Not applicable.

## CONSENT

Written informed consent was obtained from patient for publication of this case report and accompanying images. A copy of the written consent is available for review by the Editor‐in‐Chief of this journal by request.

## Data Availability

Data available on request from the authors.
